# Cross-cultural adaptation and translation of the Constant Murley Score into Arabic

**DOI:** 10.1051/sicotj/2020042

**Published:** 2020-10-20

**Authors:** Ali Maqdes, Sager Samir Hanna, Ahmed Khaled Bouhamra, Aliaa F. Khaja

**Affiliations:** 1 Al Razi Orthopedic Hospital Kuwait; 2 Upper Extremity Fellowship, Queens University Canada

**Keywords:** Arabic language, Constant score, Translation, Validity, Questionnaire, Outcome assessment

## Abstract

*Introduction*: Shoulder pain is a major disorder of the musculoskeletal system. To the best of our knowledge, there is no documentation of an Arabic version of the shoulder disability and pain measurements. Constant Murley Score (CMS) is one of the standard questionnaires for clinical practice and research. The aim of this research centred around the evaluation of the Arabic Constant Murley Score and subsequently assessing the reliability and validity in comparison to disabilities of the arm, shoulder, and hand (DASH). *Methods*: Hundred and twenty five patients took part in this research. We did the internal consistency tests with Cronbach’s alpha. Intra-correlation coefficient, convergent validity, convergent construct validity, responsiveness, and floor and ceiling effects were also calculated. *Results*: Principal component analysis showed that the variance was 63.31% with a factor range of 0.42–0.85, which fulfils the uni-dimensionality criterion. Also, the Arabic CMS correlated negatively with the DASH score (−0.82, *p* < 0.001). The Arabic version of CMS was consistent with Cronbach’s alpha of 0.74. With Inter Class Correlation Coefficient (ICC) = 0.83 it also showed a very good test-retest reliability. *Conclusion*: Ours is the first translation and cross-cultural adaptation of the CMS into Arabic. Important evidences of validity were tested such as uni-dimensionality, convergent validity, and internal consistency. Results demonstrate an acceptable Cronbach’s alpha of 0.74, ICC = 0.830 indicating excellent reliability and a strong correlation of the Arabic CMS with the DASH score (*r* = −0.820). Overall, the Arabic version of CMS is a good and reliable diagnostic tool for patients experiencing shoulder pain.

## Introduction

One of the commonest clinical defects of the musculoskeletal system is shoulder pain. Sometimes, it poses challenges in therapy and diagnosis [1]. Studies have shown that shoulder pain accounts for at least five percent of musculoskeletal consultations [2, 3]. There are several validated scores employed in shoulder assessment; in most cases, these scores are subjected to objective measurements by clinicians, and the measurement is time-consuming [4, 5]. CMS was designed for the evaluation of the prognosis after treatment of a shoulder defect [5]. CMS is reproducible, has a high sensitivity, and with a high intra and inter observability [6–9].

Patient-reported outcome measures give an insight into the patient’s perspective on how disease and its course of treatment impacts their quality of life and health. Most outcome measures for musculoskeletal malfunctions tend to be specific to joints, for instance the CMS [6]. Outcome measures can also be generic and disease-specific (SF-12 and DASH scores). Generally, outcome measures must be reliable and valid in precise measurements and indicating minimal intra observer error as well as inter observer error [8]. A high sensitivity to change is also of utmost importance.

The CMS was designed for assessment of overall value or assessment of the physiology of a treated, diseased, or normal shoulder. CMS comprises subjective and objective sections comprising four subdivisions, pain (with a maximum of 15 points), strength, activities of daily living, and motion range with maximum points of 25, 20, and 40 respectively. A high score translates to qualitative function (minimum of 0 points maximum of 100 points) [7].

Although Constant Score is widely used in Arabic nations for assessment of shoulder pathologies, translation and cultural adaptations of the modified constant score and standard test protocols are yet to be provided. Cross-cultural adaptations may enhance the comprehension of properties measured. Translations that have been validated are vital considering the increasing percentage of multinational and multicenter studies, which lends higher statistical relevance to randomized controlled trials [5, 9]. Considering the socio-economic impact and prevalence of shoulder pathologies, we opine that an Arabic adaptation and validation of constant score would immensely benefit Arab-speaking patients and surgeons.

### Disabilities of the arm shoulder and hand (DASH)

DASH questionnaire consists of 30 items. All items are self-reported and designed specifically for the measurement of symptoms and physical functions in participants experiencing musculoskeletal disorders of the upper limb. With DASH, the clinician has a first-hand view of the disability suffered by patients with malfunctioned upper limbs. They are also able to observe any changes that may occur in function or symptoms over time [10]. It proved to be a reliable tool for physicians to investigate the joints in the upper extremity. Each item has a score range of 1–5 with the total score calculated by adding the score of all rated 30 items from 30 (no disability) to 150 (highest level of disability). An Arabic translation and validation have been done by Alotaibi et al. [11]. The Arabic DASH score proved to be reliable and valid, as well as a responsive outcome measure for Arabic patients; it can be conveniently employed for documentation of the patient’s status while also offering support for clinical practice [11]. This is currently the only score translated and validated into Arabic found in the literature.

## Materials and methods

### Translation

Translation was done according to the guidelines issued by Guillemin et al. [12], Mathias et al. [13], Wild et al. [14], and Epstein et al [15]. Two orthopaedic surgeons, both bilingual, were a part of the translation panel. Also included were an Arab-speaking proof-reader, and an independent translation agency. The Constant score was initially translated to Arabic, then re-translated, and revised by the translation panel. We conducted a pilot study on 10 bilingual patients. Patients were chosen at random to fill out the questionnaire. Then we investigated for the patients’ interpretation of each item, language ease, and understanding of the concepts and assessed for need of assistance when filling out the questionnaire before proceeding to launch our full-scale investigation. We obtained permission from the original author of the CMS and was involved in all the stages of this process.

### Participants

Hundred and twenty five patients were involved in the study, and they completed both DASH and CMS questionnaires. All participants gave their consent for analysis of their respective data. The mean age was 52.4 years. The least age was 17 while the oldest was 81 years. Patients were given two patient-related outcome questionnaires adapted for Arabic speakers. The Kuwaiti Ministry of Health Ethical Committee is the main authority of patient record keeping, and they had approved this study.

### CMS questionnaire

The four constructs (ADL, pain, range of motion, and strength) of the CMS questionnaire exploratory factor analysis was done. Also, we determined how many factors were extracted via the varimax rotation and the principal component analysis [16]. We included items whose loading factor exceeded 0.4.

### Patient burden and feasibility

We recorded the average time it took each participant to fill out the questionnaire as well as if they required any assistance during the process as part of the patient-burden investigation.

The feasibility was determined by missing or incomplete responses and these entries were not included in the study.

### Data analysis and psychometric scale properties

We used Cronbach’s alpha to evaluate the internal consistency. According to the literature, *α* > 0.70 is largely acceptable, but it should not exceed 0.95 in order to avoid redundancy [17]. We assessed test-retest reliability using ICC. We measured content validity via examination of the data distribution shape, alongside ceiling and floor effects. Floor effect represents the lowest percentage score (0), while the ceiling effect is indicative of the highest percentage score (100). If the percentage of respondents with ceiling or floor effect exceeded 30, then they would be deemed to be relevant.

We investigated Spearman’s correlation coefficient between CMS and DASH. The goal was to test for the CMS convergent validity. Because Arabic DASH has been validated, a high correlation coefficient would be a major proof of CMS validity.

## Results

**Figure 1:**
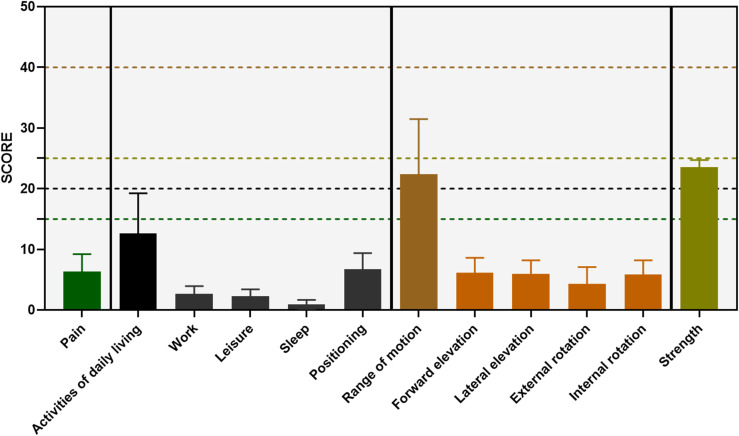
The range of Arabic CMS scores.

Hundred and twenty five patients were involved in the study, and they completed both DASH and CMS questionnaires. All participants gave their consent for analysis of their respective data. Mean age was 52.4 years with SD (Standard deviation) of 11.4 years. This implies that most of the samples were between 41 and 64 years of age. The least age was 17 while the oldest was 81. The range of Arabic CMS scores, including the constructs and their subscales are demonstrated in Figure 1 (the dashed lines and their colours indicate the maximum possible score for each construct).

For all the items, no ceiling and floor effect was recorded. We used Shapiro–Wilk test to verify if the data in Arabic CMS deviated significantly from the normal distribution, which was not the case (0.750, *p* = 0.063). A *p*-value > 0.05 is an indication of data with normal distribution.

### Uni-dimensionality

As shown in Table 1, we performed the principal component analysis (PCA) to investigate the uni-dimensionality of the questionnaire. The result obtained indicated that there was a variance value of 63.31% highlighted by the one-factor solution, which confirms that Arabic CMS fulfils the one-dimensionality criterion.

**Table 1:** Descriptive statistics and principal component analysis.

CMS items	Min	Max	Mean	SD	Cronbach’s *α*	PCA factor
Pain	0	15	6.34	2.86		0.57
Activities of daily living	2	20	12.59	6.65	0.76	
Work	0	4	2.71	1.23		0.75
Leisure	0	4	2.23	1.16		0.78
Sleep	0	2	0.92	0.73		0.56
Positioning	2	10	6.73	2.65		0.67
Range of motion	0	40	22.35	9.12	0.78	
Forward elevation	0	10	6.18	2.43		0.78
Lateral elevation	0	10	5.98	2.21		0.85
External rotation	0	10	4.32	2.75		0.42
Internal rotation	0	10	5.87	2.33		0.77
Strength	0	25	23.57	1.15		0.67

### Reliability

We estimated the CMS reliability by calculating Cronbach’s alpha which equated to 0.74, showing that the internal consistency was of a higher degree. The subscale of activities of daily living and range of motion each showed an acceptable degree of internal consistency, with Cronbach’s alpha of 0.76 and 0.78, respectively.

For the test-retest reliability, the participants completed the CMS twice at an interval of 7.50 ± 1.25 days and no significant difference was observed between the first and second assessments (*p* = 0.233). The value of the ICC was 0.83. According to the previously published study by Koo et al., values that are lower than 0.5, within the range of 0.5–0.75, and above 0.90 clearly indicates poor reliability, moderate reliability, good and excellent reliability, respectively [18]. In this case, the ICC indicates excellent reliability.

### Construct validity

By calculating the Spearman correlation test, it could demonstrate that the Arabic CMS was negatively correlated (*r* = − 0.82, *p* ≤ 0.001) with DASH score, which indicates that Arabic version of CMS is also an optimal diagnostic tool for reporting the condition of the patient’s shoulder.

## Discussion

This is the maiden translation and cultural adaptation of the CMS to Arabic. To achieve this, we tested important pieces of evidence of validity in the Arabic CMS, like internal consistency, uni-dimensionality, and convergent validity. We deduce that Arabic CMS has a high level of reliability, internal consistency, and validity to estimate the condition of the patient’s shoulders.

No ceiling effects or floor effects were displayed in the results. The items contained in the CMS questionnaire had factor loadings which exceeded 0.70 after the one-factor PCA examination. Also, an acceptable Cronbach’s alpha of 0.74 points to an internally consistent questionnaire. Results of the test-retest (ICC = 0.830) also demonstrated an excellent reliability. Also, we discovered that the Arabic version of CMS was strongly correlated with the DASH score (*r* = −0.820). The results proved the clinical usability of the Arabic version of the CMS questionnaire for evaluating patients experiencing pain in the shoulder.

The Constant score was designed as a scoring system for evaluating the prognosis of patients experiencing shoulder pathologies. However, it has received a lot of criticism due to its reliance on seemingly imprecise terminologies and the absence of standard protocol [19]. A study by Blonna et al. proved significant improvements of both intra and inter observer reliability after employing standardized Constant Score protocols and created a version without incorporating the modified guidelines issued by Constant in 2008 [7, 20]. Previously, the strength measurement in the Constant-Murley Score did not include a standardized protocol for measurement. As a result, in 2010, Hirschmann et al. proposed a standardized arm and torso position (90° of shoulder abduction without torso stabilization) to ensure reliability during strength measurement of Constant score [21].

Recent cross-cultural adaptations and modifications of the Constant Murley Score into Danish and Turkish gave rise to a standardized test protocol incorporating the modified and new guidelines issued by Constant alongside its validity and reliability assessment [22, 23]. The Danish version was validated in a study involving 45 patients between the age of 59.0 ± 17.7 years. The study involved the analysis of ceiling effects, floor effects, agreement, inter-rater and intra-rater reliability. However, they did not have strong analytical power because of the small sample size. In the Arabic study of CMS, 125 patients have participated which gives much better power to the current study. By comparing with Moeller et al., we think that the results presented in our study is a more reliable CMS cross-cultural adaptation [24].

Adaptation and translation into the Greek language was done for the Constant Murley Score in 2017. Ntourantonis et al. had 63 patients in their study [25]. Cronbach alpha was found to be 0.92, test-retest reliability of 0.95 and construct validity between quick-DASH and CMS was 0.84 [25]. They concluded that the Greek version of CMS was reliable and found to be closely related to the original English CMS. Similar results were found in the Arabic translations of other patient-related outcome regional scores. For instance, the Arabic translation of the Early Onset Scoliosis Questionnaire-24, had reported a Cronbach’s alpha test of 0.919, confirming excellent reliability [26]. Another study compared the reliability of Knee scores translated into Arabic and reported that the Arabic International Knee Documentation Committee Subjective Knee Form had an excellent Cronbach’s alpha test of 0.95. Whereas, the Arabic Lysholm Knee Score and Oxford Knee Score versions had Cronbach’s alpha tests of 0.8 and 0.85, respectively [27] (see Supplementary Material 1).

Equally important is that surgeons have to keep in mind and carefully choose which patients can be given the CMS, according to pathology. In a systematic review for evaluation of the CMS psychometric properties in pathologies of the shoulder, it is valid for use in subacromial pathology [28]. While other pathologies like arthritis, fractures, frozen shoulder, and instability showed inconclusive results.

## Conclusion

Although the Constant Score has been heavily criticized, it is still employed for the assessment of the functional status of patients experiencing shoulder pathologies. We successfully translated and adapted the CMS into Arabic. It is important to recognize that the Arabic CMS can help in the evaluation of shoulder pathologies among Arabic-speaking surgeons and patients. At the same time, excellent results concerning construct validity, reliability, and internal consistency can be maintained. Future steps could focus on confirmation of the responsiveness of our Arabic version of the modified CMS.

## Supplementary material

Supplementary material is available at https://www.sicotj-journal.org/10.1051/sicotj/2020042/olm. *Supplementary Material 1*. Arabic version of constant score.

## Declarations

### Competing interests

None.

### Ethical approval & publishing consent

- We obtained ethical approval.
- We obtained consent to publish from the participants.
- Name of Ethical Committee: Ministry of Health, Kuwait, Research and publication office.
- Committee Reference Number: 2019/1060.

### Consent to publish

Consent of participation and publication was obtained with written format from all participants.

### Availability of data and material

Data supporting these findings were obtained from the Ministry of Health Al Razi Orthopedic Hospital, Kuwait. However, the ability of the data used in this study are restricted, and so are not publicized. However, these data can be obtained from the authors if a request is made and permission obtained from the Ministry of Health Al-Razi Orthopedic Hospital, Kuwait.

### Authors’ contributions

Equal contributions were made by all authors.
