# Supplementary material for: Cross-cultural adaptation and translation of the Constant Murley Score into Arabic
Source: SICOT J. 2020 Oct 20;6:44. doi: 10.1051/sicotj/2020042 (PMC7680506; doi:10.1051/sicotj/2020042)
Supplement: Supplementary file 1 — Supplementary Material 1. Arabic version of constant score. [file sicotj-6-44-s1.pdf]

تقييم كونستانت للكتف
Constant Shoulder Score

Clinician's name (or ref)_______________________ **Patient's name (or ref)** _______________________

متعددة الاختيارات التالية، يرجى اختيار جواباً واحداً ما إن تم تحديد غير ذلك. لرجاء الإجابة عن جميع الأسئلة
Answer all questions, selecting just one unless otherwise stated.

خلال الأربعة أسابيع الماضية
During the past 4 weeks

ألم<br>1.Pain	
لا يوجد ألم<br>None	☐
طفيف<br>Mild	☐
معتدل<br>Moderate	☐
حاد<br>Severe pain	☐

٢. مستوى النشاط (اختر كل ما ينطبق)<br>2. Activity Level (check all that apply)	
القدرة على أداء العمل<br>Ability to work	☐
القدرة على أداء الأنشطة الترفيهية<br>Ability to engage in recreational activities	☐
القدرة على النوم<br>Ability to sleep	☐

٣. وضعية الذراع<br>3. Arm Positioning	
الخصر<br>Waist	☐
الصدر<br>Chest	☐
الرقبة<br>Neck	☐
الرأس<br>Head	☐
فوق الرأس<br>Above head	☐

مجموع المريض ☐
Patient score
